# Editorial: Recent Advances in Crustacean Endocrinology

**DOI:** 10.3389/fendo.2021.730642

**Published:** 2021-07-15

**Authors:** Haihui Ye, Marcy N. Wilder, Heinrich Dircksen, J. Sook Chung

**Affiliations:** ^1^ College of Fisheries, Jimei University, Xiamen, China; ^2^ Fisheries Division, Japan International Research Center for Agricultural Sciences, Tsukuba, Japan; ^3^ Department of Zoology, Stockholm University, Stockholm, Sweden; ^4^ Institute of Marine and Environmental Technology, University of Maryland Center for Environmental Science, Baltimore, MD, United States

**Keywords:** reproduction, metabolic regulation, growth, genetics, neuropeptides, Crustacea

Crustacea constitute an important taxonomic group found throughout global aquatic ecosystems; their various physiological processes and life functions are regulated by the crustacean endocrine system working in concert with the nervous system. The objective of this Special Issue was to provide a forum for researchers to report upon cutting-edge research in Crustacean Endocrinology carried out using a variety of experimental models. This Research Topic contains 15 contributions, comprising eight original research articles, two brief research reports, and five reviews.

At the outset, crustacean eyestalk hormones are of great interest due to their key roles in controlling multiple physiological processes, among these, metabolic function, osmoregulation, molting, and reproduction. In this Special Issue, Meng et al. reported that in the swimming crab *Portunus trituberculatus*, ovarian proteome and miRNA profiles were altered following eyestalk ablation, and that miRNA-protein network analysis suggested that miRNAs are involved in promoting ovarian maturation by controlling the expression of proteins related to methyl farnesoate synthesis, calcium signaling, and energy metabolism. Further regarding *P. trituberculatus*, Jiang et al. found that the insulin receptor (Pt-IR) and the insulin-like growth factor-binding protein (Pt-IGFBP) may be the targets of eyestalk neuropeptides and thus respond to eyestalk ablation independently from insulin-like androgenic gland (IAG) hormone regulation. Kang et al. employed the whiteleg shrimp, *Litopenaeus vannamei*, and injected dsRNA corresponding to multiple sinus gland peptides (SGPs) into subadults; it was found that expression was significantly decreased for *SGP-G*, the most predominant isoform expressed in the eyestalks, while vitellogenin (Vg) gene expression in the ovaries and concentrations of Vg protein in the hemolymph were not changed by this treatment. In the mud crab *Scylla paramamosain*, Liao et al. investigated the transcriptional regulation of Vih (*SpVih*) and revealed that the binding site of Oct4/Sox9 transcription factor may be the key region for the positive regulation of the expression of *SpVih*. Also of interest, crustacean female sex hormone (CFSH) is a key regulator of crustacean sexual differentiation. Jiang et al. demonstrated that *Sp-CFSH* is expressed exclusively in the eyestalks in *S. paramamosain*, and that DNA methylation inhibits *Sp-CFSH* expression by blocking the binding of transcription factor Sp1. Sun et al. analyzed the eyestalk transcriptome of the oriental river prawn, *Macrobrachium nipponense* during salinity acclimation and found that 1,392 and 1,409 genes were differentially expressed in the eyestalks in response to conditions of low and high salinity. In a review by Chen et al. on the crustacean hyperglycemic hormone superfamily, the identification of the first receptors functioning for ion transport peptides (ITPs) in the silkworm as insect members of this superfamily was highlighted; this content is expected to provide impetus to other researchers to conduct further functional studies on these peptides in the near future.

In addition to eyestalk neurohormones, other hormones and factors are also addressed in this Special Issue. Toyota et al. investigated the *in vivo* physiological functioning of methyl farnesoate and 20-hydroxyecdysone during the larval stages of the kuruma prawn *Marsupenaeus japonicus*, shedding light not only on the ecotoxicological impacts of insect growth regulators (IGRs), but also on endocrine mechanisms underlying larval metamorphosis in benthic decapod crustaceans. Tsutsui et al. investigated gonadal peptide hormones and peptide hormone receptors by analyzing the transcriptome of the ovary of *M. japonicus*. The ovarian transcriptome data thus generated led to the identification of five candidate peptide hormones, including bursicon-α and -β, crustacean hyperglycemic hormone (CHH)-like peptide, insulin-like peptide (ILP), and neuroparsin-like peptide (NPLP). These results further suggested that various gonadal peptide hormones akin to those in vertebrates regulate reproductive physiology in crustaceans, although the actual substances differ. Bao et al. identified a total of 61 peptide and 40 G-protein coupled receptors transcripts from the peppermint shrimp *Lysmata vittata*. Among these, both IAG hormone and CFSH were each revealed to possess two unique mature peptides, and their transcripts showed higher expression levels in the male phase than in the euhermaphrodite phase. This suggested that these sexual differentiation hormones may be involved in producing sexual characteristics rather than promoting spermatogenesis or vitellogenesis. In relation to reproduction, Wang et al. investigated the Vg gene family in *L. vannamei*, suggesting that Vg as a substance may have some relation to growth and molt-related processes in addition to serving as a source of nutrients during the reproductive process. Furthermore, regarding this topic, Jayasankar et al. commented on the roles of wide-ranging vitellogenesis-stimulating factors, and their potential molecular functioning and associated pathways in decapod crustaceans. Levy and Sagi reviewed the crustacean IAG-switch, a unique crustacean endocrine mechanism, the existence of which has become clear based on earlier discoveries of the androgenic gland and IAG hormone and of more recent IAG-switch-based manipulations. These authors moreover discussed this unique, early pan-crustacean, insulin-based, sexual differentiation control mechanism in contrast to the extensively-studied mechanisms relating to vertebrate sex steroid function.

Also in this Special Issue, Mykles reviewed the extensive signaling pathways regulating the crustacean molting gland. The Y-organs transition through four physiological states over the molting cycle; these are sequentially mediated by molt-inhibiting hormone (MIH; basal state), mechanistic Target of Rapamycin complex 1 (mTORC1; activated state), Transforming Growth Factor-beta (TGFbeta)/Activin (committed state), and ecdysteroids (repressed state). Future research should focus on the interactions of such signaling pathways that integrate physiological status with assorted environmental cues, in order to obtain a fully holistic understanding of crustacean molt control.

Finally, relating to the field of endocrine disruption, Knigge et al. presented an extensive overview of the evolution of the crustacean endocrine system, highlighting known endocrine endpoints that are targets of chemical disruption, and identifying other components of endocrine signaling that may prove to be targets of disruption. This review highlights the fact that endocrine disruption in crustaceans needs to be more deeply evaluated with respect to their unique endocrine systems, at least for those of several model crustaceans used in ecotoxicology, as they differ considerably from those of vertebrate species.

In conclusion, as Guest Editors of this Special Issue, we would like to express our sincerest thanks to all of the authors for their valuable contributions to this Research Topic, to the many reviewers who generously gave their time allowing the research contributions to be presented in their best light, and to the regular editors serving *Frontiers in Endocrinology* and the relevant journal personnel for their guidance throughout this project. We hope that this Special Issue will not only serve as useful reference material but also as a stimulus to all those involved in research on Crustacean Endocrinology.

## Author Contributions

HY wrote the draft. MW, HD, and JC revised the text. All authors contributed to the article and approved the submitted version.

## Conflict of Interest

The authors declare that this editorial was written in the absence of any commercial or financial relationships that could be construed as a potential conflict of interest.

